# Supplementary figures and images for: Bumblebees acquire alternative puzzle-box solutions via social learning
Source: PLoS Biol. 2023 Mar 7;21(3):e3002019. doi: 10.1371/journal.pbio.3002019 (PMC9990933; doi:10.1371/journal.pbio.3002019)

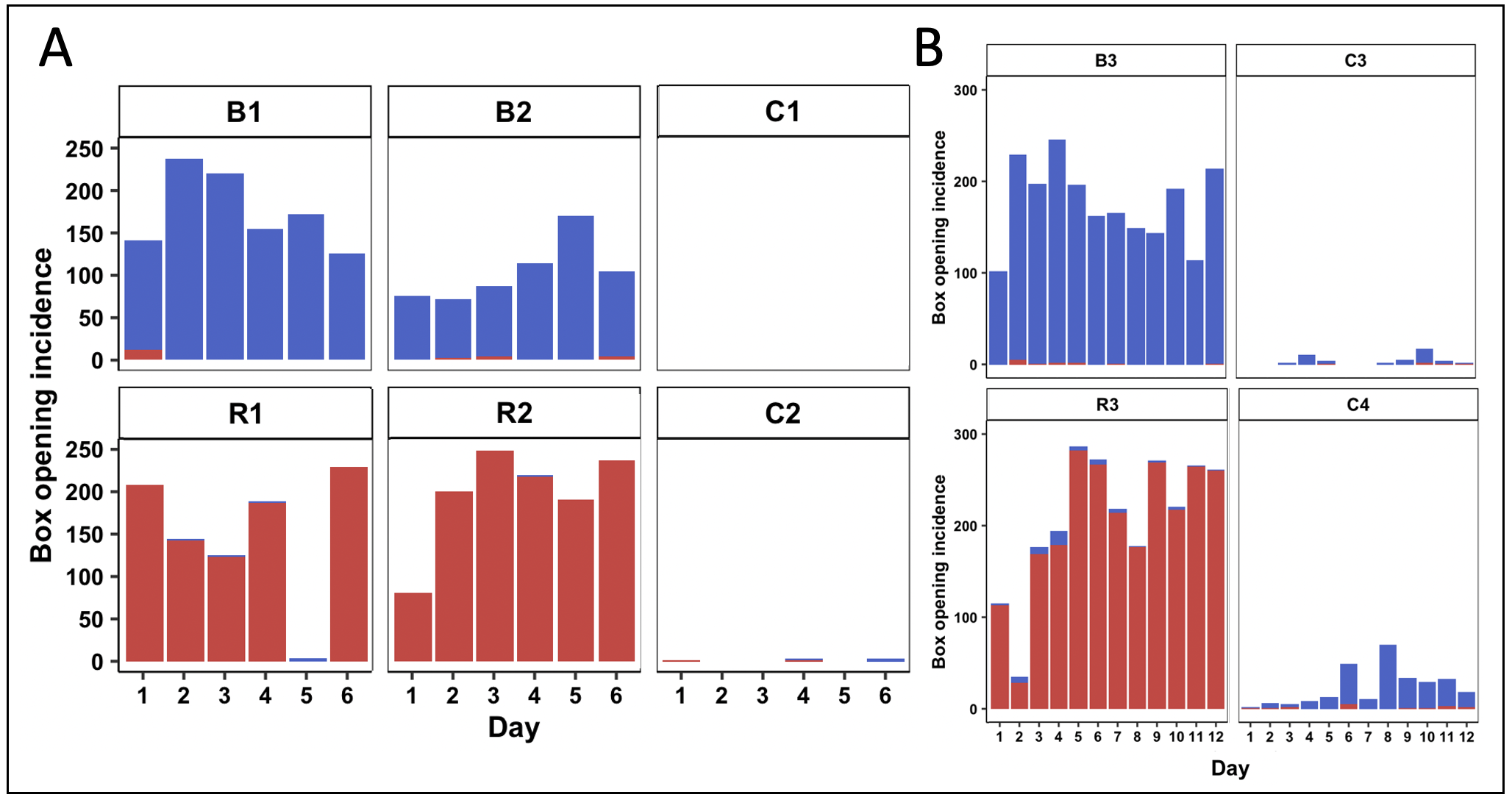

Supplement: S1 Fig — Daily overall box-opening incidence in the (A) 6-day and (B) 12-day diffusion experiments. Overall data includes incidences of box opening by the demonstrator and incidences of box opening that were not assigned to any observer ID. Colonies B1–3 were each seeded with a demonstrator trained in the blue tab/anticlockwise pushing technique. Colonies R1–3 were each seeded with a demonstrator trained in the red tab/clockwise pushing technique. Colonies C1–4 were controls that lacked a demonstrator. Incidences of the blue tab/anticlockwise pushing technique are depicted in blue, while incidences of the red tab/clockwise pushing technique are depicted in red. The data underlying this figure can be found in https://doi.org/10.6084/m9.figshare.21353973. (TIF) [file pbio.3002019.s003.tif]

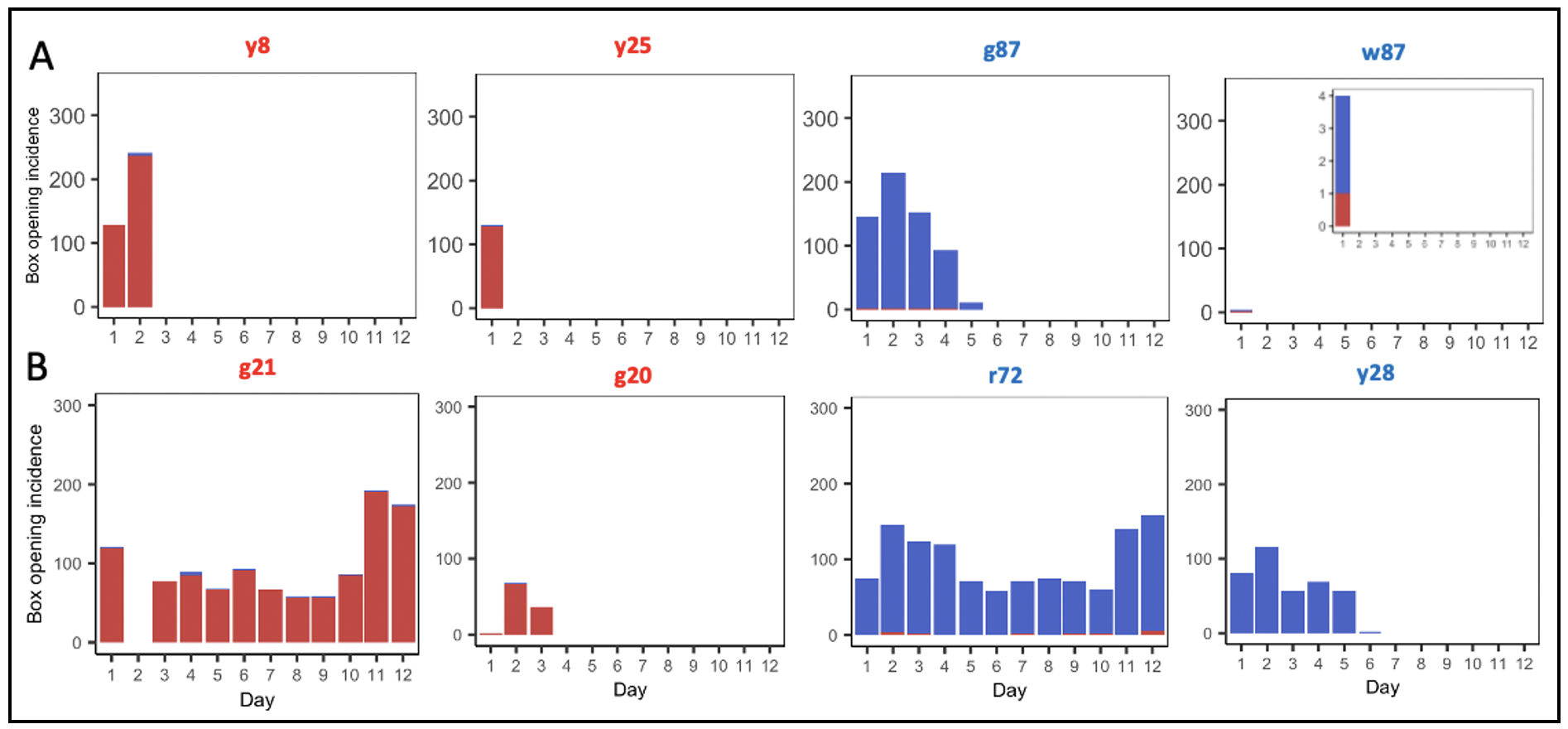

Supplement: S2 Fig — Individual box-opening incidence data for demonstrators in (A) population 1R2B2 and (B) population 2R2B2. The colour of the graph titles indicates the trained behavioural variant for each demonstrator. The inset data for w87 is the same as in the wider graph, presented at a finer scale for the sake of clarity. The data underlying this figure can be found in https://doi.org/10.6084/m9.figshare.21353973. (TIF) [file pbio.3002019.s004.tif]

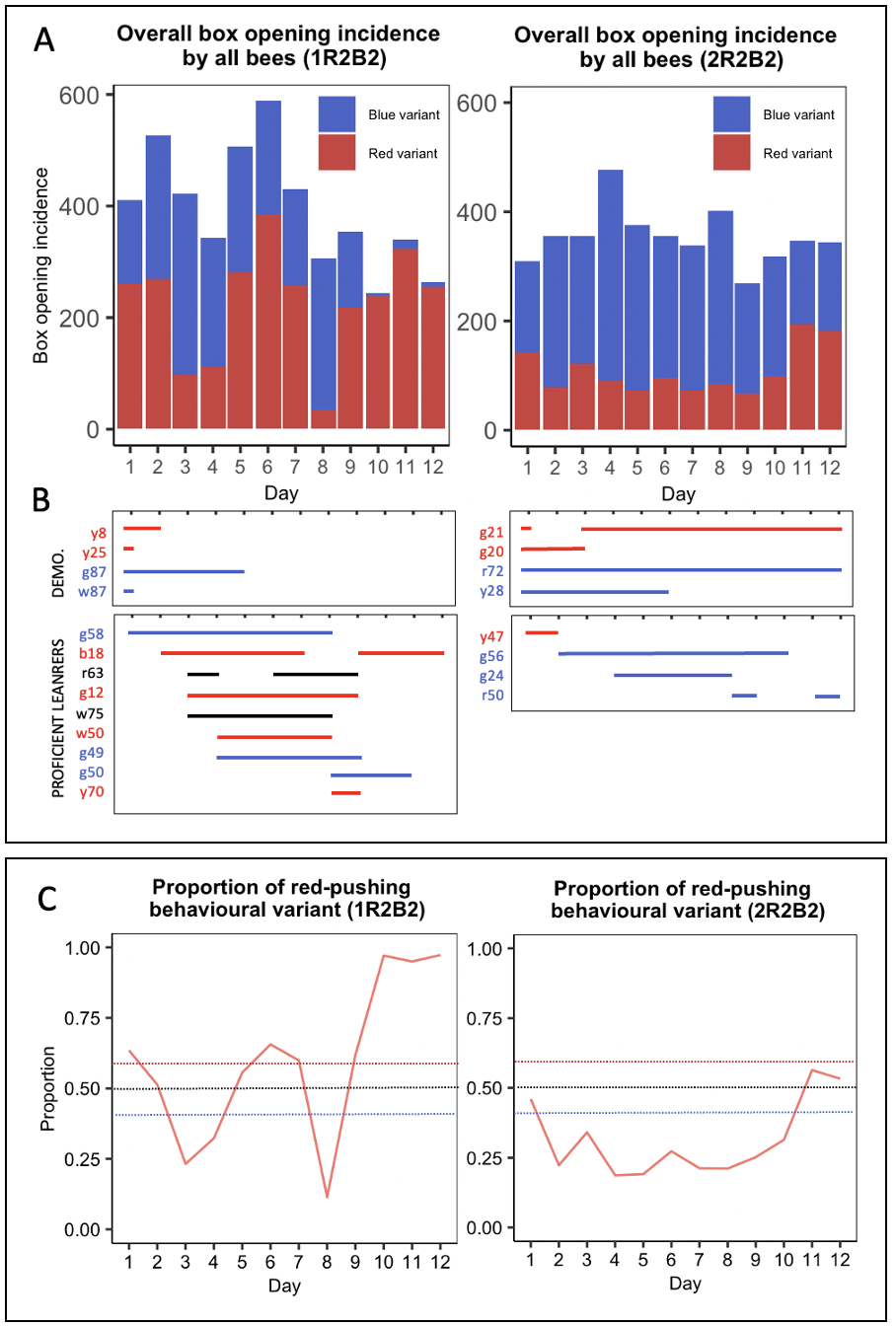

Supplement: S3 Fig — Data includes incidences of box opening by the demonstrator and incidences of box opening that were not assigned to any observer bee ID. (A) Overall box-opening incidence by all bees in Experiment 3 (left panel, population 1R2B2; right panel, population 2R2B2). The incidence of each behavioural variant is indicated by colour. (B) Days spent active by trained demonstrators and proficient learners. (C) The proportion of recorded daily behaviours that were the red-pushing variant. Dashed lines show the thresholds for a preference for either variant. The data underlying this figure can be found in https://doi.org/10.6084/m9.figshare.21353973. (TIF) [file pbio.3002019.s005.tif]
