# Supplementary material for: Bumblebees acquire alternative puzzle-box solutions via social learning
Source: PLoS Biol. 2023 Mar 7;21(3):e3002019. doi: 10.1371/journal.pbio.3002019 (PMC9990933; doi:10.1371/journal.pbio.3002019)
Supplement: S1 Table — (DOCX) [file pbio.3002019.s006.docx]

**Appendix Table 1. Total daily box opening and variant incidence (single-demonstrator diffusion experiments)**

| 1. **Demonstrator data^1^** | | | | | | | | | | | | | |  |  |  |  |
| --- | --- | --- | --- | --- | --- | --- | --- | --- | --- | --- | --- | --- | --- | --- | --- | --- | --- |
| **Colony ID** | **Box opening incidence** | | | | | | | | | | | | | **Total red variant** | **Total blue variant** | **Daily average** |  |
|  | **Day 1** | **2** | **3** | **4** | **5** | **6** | **7** | **8** | **9** | **10** | **11** | **12** | **Total** |  |  |  |  |
| **R1** | 207 | 138 | 87 | 50 | 69 | 53 | - | - | - | - | - | - | 604 | 601 | 3 | 100.67 |  |
| **R2** | 80 | 199 | 247 | 214 | 84 | 113 | - | - | - | - | - | - | 937 | 937 | 0 | 156.17 |  |
| **R3** | 114 | 35 | 170 | 180 | 202 | 139 | 126 | 100 | 87 | 74 | 104 | 93 | 1424 | 1385 | 39 | 118.67 |  |
| **B1** | 114 | 154 | 107 | 95 | 72 | 57 | - | - | - | - | - | - | 599 | 0 | 599 | 99.83 |  |
| **B2** | 75 | 72 | 80 | 93 | 67 | 47 | - | - | - | - | - | - | 434 | 9 | 425 | 72.33 |  |
| **B3** | 102 | 221 | 194 | 241 | 193 | 154 | 1 | 0 | 0 | 0 | 0 | 0 | 1106 | 3 | 1103 | 92.17 |  |
| **C1** | n/a | n/a | n/a | n/a | n/a | n/a | n/a | n/a | n/a | n/a | n/a | n/a | n/a | n/a | n/a | n/a |  |
| **C2** | n/a | n/a | n/a | n/a | n/a | n/a | n/a | n/a | n/a | n/a | n/a | n/a | n/a | n/a | n/a | n/a |  |
| **C3** | n/a | n/a | n/a | n/a | n/a | n/a | n/a | n/a | n/a | n/a | n/a | n/a | n/a | n/a | n/a | n/a |  |
| **C4** | n/a | n/a | n/a | n/a | n/a | n/a | n/a | n/a | n/a | n/a | n/a | n/a | n/a | n/a | n/a | n/a |  |
| 1. **Observer data only^2^** | | | | | | | | | | | | | | | | |  |
| **Colony ID** | **Box opening incidence** | | | | | | | | | | | | | **Total red variant** | **Total blue variant** | **r_s_^3^** | **p** |
|  | **Day 1** | **2** | **3** | **4** | **5** | **6** | **7** | **8** | **9** | **10** | **11** | **12** | **Total** |  |  |  |  |
| **R1** | 0 | 1 | 35 | 138 | 220 | 171 | - | - | - | - | - | - | 565 | 559 | 6 | 0.94 | 0.017^*^ |
| **R2** | 0 | 0 | 0 | 4 | 103 | 116 | - | - | - | - | - | - | 223 | 222 | 1 | 0.94 | 0.005^**^ |
| **R3** | 1 | 0 | 5 | 9 | 83 | 124 | 89 | 68 | 174 | 142 | 154 | 160 | 1009 | 993 | 16 | 0.90 | <0.001^***^ |
| **B1** | 28 | 83 | 111 | 52 | 100 | 63 | - | - | - | - | - | - | 437 | 14 | 423 | 0.26 | 0.658 |
| **B2** | 0 | 0 | 5 | 19 | 101 | 57 | - | - | - | - | - | - | 182 | 2 | 180 | 0.93 | 0.008^**^ |
| **B3** | 0 | 8 | 1 | 3 | 1 | 3 | 164 | 149 | 143 | 191 | 109 | 209 | 980 | 9 | 971 | 0.81 | 0.001^**^ |
| **C1** | 0 | 0 | 0 | 0 | 0 | 0 | - | - | - | - | - | - | 0 | 0 | 0 | - | - |
| **C2** | 0 | 0 | 0 | 3 | 0 | 2 | - | - | - | - | - | - | 5 | 2 | 3 | 0.54 | 0.268 |
| **C3** | 0 | 0 | 2 | 11 | 3 | 0 | 0 | 0 | 4 | 16 | 4 | 1 | 41 | 4 | 37 | 0.38 | 0.226 |
| **C4** | 2 | 4 | 5 | 8 | 13 | 49 | 8 | 70 | 33 | 27 | 32 | 17 | 269 | 16 | 253 | 0.71 | 0.010^**^ |

^1^Demonstrator data includes incidences of box opening by trained demonstrators only. ^2^Box openings were assigned to observers only when they pushed a tab ≥50% of the required distance to open the box; and obtained the reward. ^3^For the 6-day diffusions, df=4. For the 12-day diffusions, df=10. ^*^p<0.05, ^**^p<0.01 and ^***^p<0.001. Data were analysed using Spearman’s rank order correlation tests.
