# Supplementary material for: Bumblebees acquire alternative puzzle-box solutions via social learning
Source: PLoS Biol. 2023 Mar 7;21(3):e3002019. doi: 10.1371/journal.pbio.3002019 (PMC9990933; doi:10.1371/journal.pbio.3002019)
Supplement: S2 Table — (DOCX) [file pbio.3002019.s007.docx]

**Appendix Table 2. Results of linear mixed-effects model to assess the effect of demonstrator presence on learning proficiency over time**

| **A.** | | | | | | | |
| --- | --- | --- | --- | --- | --- | --- | --- |
| **Model** | **Random factor: Bee ID** | | | | | | |
| **Code** | responsevar ~ day.f * treatment.f + (1 \| beeID.f) | | | | | | |
|  | | **Sum Sq** | **Mean Sq** | **NumDF** | **DenDF** | **F value** | **Pr(>F)** |
| **Day.f** | | 6707.2 | 6707.2 | 1 | 31.673 | 12.143 | 0.001465** |
| **Treatment.f** | | 5749.5 | 5749.5 | 1 | 29.558 | 10.40 | 0.003059** |
| **Day.f:treatment.f** | | 5607.3 | 5607.3 | 1 | 31.673 | 10.152 | 0.003233** |
| **AIC** | | 532.86^#^ |  |  |  |  |  |
| **B.** | | | | | | | |
| **Model** | **Random factor: Colony ID** | | | | | | |
| **Code** | responsevar~day.f*treatment.f+(1\|colonyID.f) | | | | | | |
|  | | **Sum Sq** | **Mean Sq** | **NumDF** | **DenDF** | **F value** | **Pr(>F)** |
| **Day.f** | | 7247.4 | 7247.4 | 1 | 45.344 | 10.1120 | 0.002656** |
| **Treatment.f** | | 5656.3 | 5656.3 | 1 | 3.971 | 7.8920 | 0.048756* |
| **Day.f:treatment.f** | | 6096.0 | 6096.0 | 1 | 45.344 | 8.5055 | 0.005488** |
| **AIC** | | 534.76 |  |  |  |  |  |
| **C.** | | | | | | | |
| **Model** | **Random factor: Bee ID nested within Colony ID** | | | | | | |
| **Code** | responsevar~day.f*treatment.f+(1\|colonyID.f/beeID.f) | | | | | | |
|  | | **Sum Sq** | **Mean Sq** | **NumDF** | **DenDF** | **F value** | **Pr(>F)** |
| **Day.f** | | 7247.4 | 7247.4 | 1 | 27.000 | 11.2838 | 0.002342** |
| **Treatment.f** | | 5251.3 | 5251.3 | 1 | 3.133 | 8.1759 | 0.061374 |
| **Day.f:treatment.f** | | 6096.0 | 6096.0 | 1 | 27.000 | 9.4911 | 0.004709** |
| **AIC** | | 536.41 |  |  |  |  |  |

**P<0.01 and *P<0.05. ^#^The selected model used for analysis, with the lowest AIC. There were two categorical fixed effects: one between-subjects factor “treatment” (experimental, control) and one within-subjects factor “day” (day 1, day 3; where day 1 was the day an individual met the learning criteria and day 3 was two days following this). Individuals that learned too late in the diffusion to have any data recorded for day 3 were excluded (e.g. a bee that met criteria on day 5 or 6 in the 6-day diffusion experiments, or day 11 or 12 of the 12-day diffusion experiments; n=4 from the experimental colonies and n=3 from the control colonies, leaving n=18 and n=11 in each group, respectively). The response variable was box opening incidence.
