# Supplementary material for: Bumblebees acquire alternative puzzle-box solutions via social learning
Source: PLoS Biol. 2023 Mar 7;21(3):e3002019. doi: 10.1371/journal.pbio.3002019 (PMC9990933; doi:10.1371/journal.pbio.3002019)
Supplement: S3 Table — (DOCX) [file pbio.3002019.s008.docx]

**Appendix Table 3. Daily box opening incidence by individual observers (single-demonstrator 6-day diffusion experiments)**

| **Bee ID** | **Colony ID** | **Day learning criteria met** | **Box opening incidence** | | | | | | | **Total red variant** | **Total blue variant** | **Individual learner proficiency index** |
| --- | --- | --- | --- | --- | --- | --- | --- | --- | --- | --- | --- | --- |
|  |  |  | **Day 1** | **2** | **3** | **4** | **5** | **6** | **Total** |  |  |  |
| **w25** | **B1** | 1 | 25 | 81 | 81 | 0 | 8 | 4 | 199 | 14 | 185 | 33.17 |
| **w31** | **B1** | n/a | 1 | 0 | 0 | 0 | 0 | 0 | 1 | 0 | 1 | n/a |
| **w18** | **B1** | 2 | 1 | 1 | 29 | 41 | 42 | 30 | 144 | 0 | 144 | 28.80 |
| **g34** | **B1** | n/a | 0 | 0 | 1 | 0 | 0 | 0 | 1 | 0 | 1 | n/a |
| **y64** | **B1** | n/a | 1 | 0 | 0 | 0 | 0 | 0 | 1 | 0 | 1 | n/a |
| **w14** | **B1** | 4 | 0 | 1 | 0 | 11 | 49 | 28 | 89 | 0 | 89 | 29.67 |
| **g33** | **B1** | n/a | 0 | 0 | 0 | 0 | 1 | 0 | 1 | 0 | 1 | n/a |
| **wr** | **B1** | n/a | 0 | 0 | 0 | 0 | 0 | 1 | 1 | 0 | 1 | n/a |
| **y84** | **B2** | 6 | 0 | 0 | 0 | 0 | 1 | 1 | 2 | 0 | 2 | 2.00 |
| **y47** | **B2** | 4 | 0 | 0 | 1 | 1 | 0 | 7 | 9 | 0 | 9 | 3.00 |
| **y95** | **B2** | 4 | 0 | 0 | 0 | 4 | 0 | 0 | 4 | 0 | 4 | 1.33 |
| **g3** | **B2** | 4 | 0 | 0 | 1 | 3 | 1 | 0 | 5 | 0 | 5 | 1.67 |
| **g16** | **B2** | 3 | 0 | 0 | 2 | 11 | 99 | 49 | 161 | 2 | 159 | 40.25 |
| **y90** | **B2** | n/a | 0 | 0 | 1 | 0 | 0 | 0 | 1 | 0 | 1 | n/a |
| **y5** | **R1** | 4 | 0 | 1 | 0 | 36 | 77 | 83 | 197 | 196 | 1 | 65.67 |
| **r15** | **R1** | 3 | 0 | 0 | 35 | 100 | 120 | 29 | 284 | 280 | 4 | 71.00 |
| **y54** | **R1** | 5 | 0 | 0 | 0 | 1 | 21 | 59 | 81 | 81 | 0 | 27.00 |
| **y96** | **R1** | 5 | 0 | 0 | 0 | 1 | 2 | 0 | 3 | 2 | 1 | 1.00 |
| **y30** | **R2** | 6 | 0 | 0 | 0 | 0 | 0 | 17 | 17 | 17 | 0 | 17.00 |
| **y52** | **R2** | 4 | 0 | 0 | 0 | 3 | 103 | 96 | 202 | 201 | 1 | 67.33 |
| **r29** | **C2** | 4 | 0 | 0 | 0 | 3 | 0 | 2 | 5 | 2 | 3 | 1.67 |
