# Supplementary material for: Bumblebees acquire alternative puzzle-box solutions via social learning
Source: PLoS Biol. 2023 Mar 7;21(3):e3002019. doi: 10.1371/journal.pbio.3002019 (PMC9990933; doi:10.1371/journal.pbio.3002019)
Supplement: S4 Table — (DOCX) [file pbio.3002019.s009.docx]

**Appendix Table 4. Daily box opening incidence by individual observers (single-demonstrator 12-day diffusion experiments)**

| **Bee ID** | **Colony ID** | **Day learning criteria met** | **Box opening incidence** | | | | | | | | | | | | | **Total red variant** | **Total blue variant** | **Indiv. learner prof. index** |
| --- | --- | --- | --- | --- | --- | --- | --- | --- | --- | --- | --- | --- | --- | --- | --- | --- | --- | --- |
|  |  |  | **Day 1** | **2** | **3** | **4** | **5** | **6** | **7** | **8** | **9** | **10** | **11** | **12** | **Total** |  |  |  |
| **y47** | **B3** | 4 | 0 | 0 | 1 | 2 | 0 | 0 | 0 | 0 | 0 | 0 | 0 | 0 | 3 | 2 | 1 | 0.33 |
| **y1** | **B3** | 2 | 0 | 7 | 0 | 0 | 0 | 0 | 0 | 0 | 0 | 0 | 0 | 0 | 7 | 4 | 3 | 0.63 |
| **r14** | **B3** | n/a | 0 | 0 | 0 | 1 | 0 | 0 | 0 | 0 | 0 | 0 | 0 | 0 | 1 | 0 | 1 | n/a |
| **r24** | **B3** | n/a | 0 | 0 | 0 | 0 | 0 | 0 | 0 | 0 | 1 | 0 | 0 | 0 | 1 | 0 | 1 | n/a |
| **r35** | **B3** | 6 | 0 | 0 | 0 | 0 | 0 | 3 | 164 | 149 | 139 | 120 | 38 | 156 | 769 | 1 | 768 | 109.86 |
| **r27** | **B3** | n/a | 0 | 0 | 0 | 0 | 1 | 0 | 0 | 0 | 0 | 0 | 0 | 0 | 1 | 1 | 0 | n/a |
| **y40** | **B3** | 9 | 0 | 0 | 0 | 0 | 0 | 0 | 0 | 0 | 3 | 71 | 71 | 52 | 197 | 0 | 197 | 49.25 |
| **b11** | **B3** | n/a | 0 | 0 | 0 | 0 | 0 | 0 | 0 | 0 | 0 | 0 | 0 | 1 | 1 | 1 | 0 | n/a |
| **w51** | **R3** | n/a | 1 | 0 | 0 | 0 | 0 | 0 | 0 | 0 | 0 | 0 | 0 | 0 | 1 | 1 | 0 | n/a |
| **w49** | **R3** | 3 | 0 | 0 | 4 | 3 | 22 | 30 | 0 | 0 | 0 | 0 | 0 | 0 | 59 | 56 | 3 | 5.90 |
| **g93** | **R3** | 7 | 0 | 0 | 0 | 0 | 0 | 1 | 2 | 0 | 5 | 8 | 1 | 5 | 22 | 22 | 0 | 3.67 |
| **w35** | **R3** | 4 | 0 | 0 | 0 | 6 | 59 | 93 | 87 | 59 | 79 | 77 | 69 | 73 | 602 | 591 | 11 | 66.89 |
| **g56** | **R3** | 8 | 0 | 0 | 0 | 0 | 0 | 0 | 0 | 9 | 90 | 57 | 84 | 82 | 322 | 320 | 2 | 64.40 |
| **w57** | **C3** | 3 | 0 | 0 | 2 | 11 | 0 | 0 | 0 | 0 | 0 | 0 | 0 | 0 | 13 | 0 | 13 | 1.30 |
| **r24** | **C3** | 5 | 0 | 0 | 0 | 0 | 3 | 0 | 0 | 0 | 0 | 0 | 0 | 0 | 3 | 0 | 3 | 0.38 |
| **b5** | **C3** | 9 | 0 | 0 | 0 | 0 | 0 | 0 | 0 | 0 | 2 | 16 | 3 | 1 | 22 | 4 | 18 | 5.50 |
| **r42** | **C3** | 9 | 0 | 0 | 0 | 0 | 0 | 0 | 0 | 0 | 2 | 0 | 1 | 0 | 3 | 0 | 3 | 0.75 |
| **g91** | **C4** | 2 | 1 | 1 | 0 | 0 | 0 | 0 | 0 | 0 | 0 | 0 | 0 | 0 | 2 | 1 | 1 | 0.18 |
| **g63** | **C4** | 10 | 1 | 0 | 0 | 0 | 0 | 0 | 0 | 0 | 0 | 1 | 0 | 0 | 2 | 2 | 0 | 0.67 |
| **g26** | **C4** | 2 | 0 | 3 | 0 | 0 | 0 | 0 | 0 | 0 | 0 | 0 | 0 | 0 | 3 | 0 | 3 | 0.27 |
| **y46** | **C4** | 3 | 0 | 0 | 4 | 3 | 6 | 0 | 1 | 0 | 1 | 0 | 0 | 0 | 15 | 5 | 10 | 1.50 |
| **y28** | **C4** | n/a | 0 | 0 | 1 | 0 | 0 | 0 | 0 | 0 | 0 | 0 | 0 | 0 | 1 | 0 | 1 | n/a |
| **y47** | **C4** | n/a | 0 | 0 | 0 | 1 | 0 | 0 | 0 | 0 | 0 | 0 | 0 | 0 | 1 | 0 | 1 | n/a |
| **y12** | **C4** | 4 | 0 | 0 | 0 | 7 | 10 | 39 | 8 | 68 | 31 | 25 | 21 | 7 | 216 | 2 | 214 | 24.00 |
| **g40** | **C4** | n/a | 0 | 0 | 0 | 0 | 0 | 1 | 0 | 0 | 0 | 0 | 0 | 0 | 1 | 0 | 1 | n/a |
| **g41** | **C4** | 6 | 0 | 0 | 0 | 0 | 0 | 3 | 0 | 1 | 0 | 1 | 0 | 1 | 6 | 1 | 5 | 0.86 |
| **y52** | **C4** | n/a | 0 | 0 | 0 | 0 | 0 | 0 | 0 | 0 | 0 | 0 | 0 | 1 | 1 | 0 | 1 | n/a |
| **00** | **C4** | n/a | 0 | 0 | 0 | 0 | 0 | 0 | 0 | 0 | 1 | 0 | 0 | 0 | 1 | 0 | 1 | n/a |
| **g24** | **C4** | 11 | 0 | 0 | 0 | 0 | 0 | 0 | 0 | 0 | 1 | 0 | 3 | 6 | 10 | 2 | 8 | 5.00 |
| **y1** | **C4** | 11 | 0 | 0 | 0 | 0 | 0 | 0 | 0 | 0 | 0 | 0 | 2 | 0 | 2 | 1 | 1 | 1.00 |
| **g18** | **C4** | 11 | 0 | 0 | 0 | 0 | 0 | 0 | 0 | 0 | 0 | 0 | 6 | 2 | 8 | 2 | 6 | 4.00 |
