# Supplementary material for: Bumblebees acquire alternative puzzle-box solutions via social learning
Source: PLoS Biol. 2023 Mar 7;21(3):e3002019. doi: 10.1371/journal.pbio.3002019 (PMC9990933; doi:10.1371/journal.pbio.3002019)
Supplement: S5 Table — (DOCX) [file pbio.3002019.s010.docx]

**Appendix Table 5. Demonstrator characteristics in the multiple-demonstrator diffusion experiments**

| **Bee ID** | **Trained variant** | **Total incidence** | **Total red variant incidence** | **Total blue variant incidence** | **% Red variant** | **% Blue variant** | **Activity period (days)** |
| --- | --- | --- | --- | --- | --- | --- | --- |
| ***Population 1R2B2*** | | | | | | | |
| **y8** | **R** | 369 | 366 | 3 | 99.19 | 0.81 | 1-2 |
| **y25** | **R** | 131 | 129 | 2 | 98.47 | 1.53 | 1 |
| **g87** | **B** | 615 | 7 | 608 | 1.14 | 98.86 | 1-5 |
| **w87** | **B** | 4 | 1 | 3 | 25.00 | 75.00 | 1 |
|  | ***Overall*** | *1119* | *503* | *616* | *44.95* | *55.05* |  |
| ***Population 2R2B2*** | | | | | | | |
| **g21** | **R** | 1079 | 1067 | 12 | 98.9 | 1.1 | 1, 3-12 |
| **g20** | **R** | 106 | 105 | 1 | 99.1 | 0.9 | 1-3 |
| **r72** | **B** | 1165 | 17 | 1148 | 1.5 | 98.5 | 1-12 |
| **y28** | **B** | 380 | 0 | 380 | 0.0 | 100.0 | 1-6 |
|  | ***Overall*** | *2730* | *1189* | *1541* | *43.6* | *56.4* |  |
