# Supplementary material for: Bumblebees acquire alternative puzzle-box solutions via social learning
Source: PLoS Biol. 2023 Mar 7;21(3):e3002019. doi: 10.1371/journal.pbio.3002019 (PMC9990933; doi:10.1371/journal.pbio.3002019)
Supplement: S6 Table — (DOCX) [file pbio.3002019.s011.docx]

**Appendix Table 6. Total daily box opening and variant incidence (multiple-demonstrator diffusion experiments)**

| 1. **Population 1R2B2** | | | | | | | |  |  | 1. **Population 2R2B2** | | | | | | | |
| --- | --- | --- | --- | --- | --- | --- | --- | --- | --- | --- | --- | --- | --- | --- | --- | --- | --- |
| ***All bees (including demonstrators)*** | | | | | | | |  |  | ***All bees (including demonstrators)*** | | | | | | | |
| **Day** |  |  | **Incidence of box opening behaviour** | | | | |  |  | **Day** |  |  | **Incidence of box opening behaviour** | | | | |
|  |  |  | **Total** | **Red variant** | **Blue variant** | **% Red variant** | **% Blue variant** |  |  |  |  |  | **Total** | **Red variant** | **Blue variant** | **% Red variant** | **% Blue variant** |
| **1** |  |  | 410 | 261 | 149 | 63.5 | 36.5 |  |  | **1** |  |  | 309 | 142 | 167 | 46.0 | 54.0 |
| **2** |  |  | 527 | 268 | 259 | 51.3 | 48.7 |  |  | **2** |  |  | 355 | 79 | 276 | 22.3 | 77.7 |
| **3** |  |  | 422 | 98 | 324 | 23.2 | 76.8 |  |  | **3** |  |  | 355 | 121 | 234 | 34.1 | 65.9 |
| **4** |  |  | 344 | 111 | 233 | 32.3 | 67.7 |  |  | **4** |  |  | 476 | 89 | 387 | 18.7 | 81.3 |
| **5** |  |  | 507 | 282 | 225 | 55.6 | 44.4 |  |  | **5** |  |  | 377 | 72 | 305 | 19.1 | 80.9 |
| **6** |  |  | 588 | 386 | 202 | 65.6 | 34.4 |  |  | **6** |  |  | 355 | 97 | 258 | 27.3 | 72.7 |
| **7** |  |  | 431 | 258 | 173 | 59.9 | 40.1 |  |  | **7** |  |  | 339 | 72 | 267 | 21.2 | 78.8 |
| **8** |  |  | 307 | 35 | 272 | 11.4 | 88.6 |  |  | **8** |  |  | 402 | 85 | 317 | 21.1 | 78.9 |
| **9** |  |  | 354 | 219 | 135 | 61.9 | 38.1 |  |  | **9** |  |  | 270 | 68 | 202 | 25.2 | 74.8 |
| **10** |  |  | 245 | 238 | 7 | 97.1 | 2.9 |  |  | **10** |  |  | 318 | 100 | 218 | 31.4 | 68.6 |
| **11** |  |  | 340 | 323 | 17 | 95.0 | 5.0 |  |  | **11** |  |  | 346 | 195 | 151 | 56.4 | 43.6 |
| **12** |  |  | 263 | 256 | 7 | 97.3 | 2.7 |  |  | **12** |  |  | 345 | 184 | 161 | 53.3 | 46.7 |
| ***Total*** | | | *4738* | *2735* | *2003* | *57.7* | *42.3* |  |  | ***Total*** | | | *4247* | *1304* | *2943* | *30.7* | *69.3* |
| ***Demonstrator total*** | | | *1119* | *503* | *616* | *45.0* | *55.0* |  |  | ***Demonstrator total*** | | | *2730* | *1189* | *1541* | *43.6* | *56.4* |
|  |  |  |  |  |  |  |  |  |  |  |  |  |  |  |  |  |  |
|  | | | | | | | |  |  |  | | | | | | | |
| ***Untrained bees only*** | | |  |  |  |  |  |  |  | ***Untrained bees only*** | | |  |  |  |  |  |
| **Day** | **New learners** | **Cum. learners** | **Incidence of box opening behaviour** | | | | |  |  | **Day** | **New learners** | **Cum. learners** | **Incidence of box opening behaviour** | | | | |
|  |  |  | **Total** | **Red variant** | **Blue variant** | **% Red variant** | **% Blue variant** |  |  |  |  |  | **Total** | **Red variant** | **Blue variant** | **% Red variant** | **% Blue variant** |
| **1** | 0 | 0 | 0 | 0 | 0 | n/a | n/a |  |  | **1** | 1 | 1 | 18 | 16 | 2 | 88.9 | 11.1 |
| **2** | 3 | 3 | 72 | 28 | 44 | 38.9 | 61.1 |  |  | **2** | 1 | 2 | 11 | 6 | 5 | 54.5 | 45.5 |
| **3** | 1 | 4 | 270 | 97 | 173 | 35.9 | 64.1 |  |  | **3** | 1 | 3 | 55 | 4 | 51 | 7.3 | 92.7 |
| **4** | 1 | 5 | 252 | 110 | 142 | 43.7 | 56.3 |  |  | **4** | 2 | 5 | 186 | 1 | 185 | 0.5 | 99.5 |
| **5** | 2 | 7 | 497 | 282 | 215 | 56.7 | 43.3 |  |  | **5** | 1 | 6 | 173 | 5 | 168 | 2.9 | 97.1 |
| **6** | 2 | 9 | 588 | 386 | 202 | 65.6 | 34.4 |  |  | **6** | 0 | 6 | 184 | 3 | 181 | 1.6 | 98.4 |
| **7** | 1 | 10 | 431 | 258 | 173 | 59.9 | 40.1 |  |  | **7** | 0 | 6 | 193 | 3 | 190 | 1.6 | 98.4 |
| **8** | 5 | 15 | 307 | 35 | 272 | 11.4 | 88.6 |  |  | **8** | 1 | 7 | 202 | 5 | 197 | 2.5 | 97.5 |
| **9** | 1 | 16 | 354 | 219 | 135 | 61.9 | 38.1 |  |  | **9** | 0 | 7 | 124 | 3 | 121 | 2.5 | 97.5 |
| **10** | 0 | 16 | 245 | 238 | 7 | 97.1 | 2.9 |  |  | **10** | 0 | 7 | 116 | 0 | 116 | 0.0 | 100.0 |
| **11** | 0 | 16 | 340 | 323 | 17 | 95.0 | 5.0 |  |  | **11** | 0 | 7 | 4 | 1 | 3 | 25.0 | 75.0 |
| **12** | 2 | 18 | 263 | 256 | 7 | 97.3 | 2.7 |  |  | **12** | 1 | 8 | 8 | 2 | 6 | 25.0 | 75.0 |
| ***Total*** | | | *3619* | *2232* | *1387* | *61.7* | *38.3* |  |  | ***Total*** | | | *1274* | *49* | *1225* | *3.8* | *96.2* |
|  |  |  |  |  |  |  |  |  |  |  |  |  |  |  |  |  |  |

^Data for all bees is inclusive of non-complete openings. Data for untrained bees and demonstrators only does not include non-complete openings.^
