# Supplementary material for: Bumblebees acquire alternative puzzle-box solutions via social learning
Source: PLoS Biol. 2023 Mar 7;21(3):e3002019. doi: 10.1371/journal.pbio.3002019 (PMC9990933; doi:10.1371/journal.pbio.3002019)
Supplement: S7 Table — (DOCX) [file pbio.3002019.s012.docx]

**Appendix Table 7. Observer characteristics (multiple-demonstrator diffusion experiments)**

| **Bee ID** | | **Population ID** |  |  | **Day of first opening** | **Day learning criteria met** | **Variant performed when first opening box** | **Variant performed when meeting criteria** | **Consistent?** | **Variant preference^1^** | **Preference strength^2^** |
| --- | --- | --- | --- | --- | --- | --- | --- | --- | --- | --- | --- |
|  | **Proficient learners (n=9)** | | | | | | | | | | |
| **g58** | | 1R2B2 |  |  | 2 | 2 | B | B | **✓** | Blue | Strong |
| **b18** | | 1R2B2 |  |  | 2 | 2 | R | R | **✓** | Red | Strong |
| **r63** | | 1R2B2 |  |  | 3 | 6 | R | B | **X** | None | n/a |
| **g12** | | 1R2B2 |  |  | 5 | 5 | R | R | **✓** | Red | Strong |
| **w75** | | 1R2B2 |  |  | 5 | 5 | B | R | **X** | None | n/a |
| **w50** | | 1R2B2 |  |  | 6 | 6 | B | R | **X** | Red | Weak |
| **g49** | | 1R2B2 |  |  | 7 | 8 | R | B | **X** | Blue | Strong |
| **g50** | | 1R2B2 |  |  | 8 | 8 | B | B | **✓** | Blue | Weak |
| **y70** | | 1R2B2 |  |  | 8 | 8 | R | R | **✓** | Red | Strong |
| **y47** | | 2R2B2 |  |  | 1 | 1 | R | B | **X** | Red | Weak |
| **g56** | | 2R2B2 |  |  | 2 | 2 | B | B | **✓** | Blue | Strong |
| **g24** | | 2R2B2 |  |  | 4 | 4 | B | B | **✓** | Blue | Strong |
| **r50** | | 2R2B2 |  |  | 8 | 8 | B | R | **X** | Blue | Weak |
|  | |  |  |  |  |  |  |  |  |  |  |
|  | **Non-proficient learners (n=9)** | | | | | | | | | | |
| **y1** | | 1R2B2 |  |  | 2 | 2 | B | R | **X** |  |  |
| **b54** | | 1R2B2 |  |  | 2 | 9 | R | B | **X** |  |  |
| **g79** | | 1R2B2 |  |  | 3 | 3 | B | B | **✓** |  |  |
| **g84** | | 1R2B2 |  |  | 4 | 4 | R | R | **✓** |  |  |
| **r59** | | 1R2B2 |  |  | 6 | 7 | B | R | **X** |  |  |
| **g3** | | 1R2B2 |  |  | 8 | 8 | B | R | **X** |  |  |
| **g54** | | 1R2B2 |  |  | 8 | 8 | B | B | **✓** |  |  |
| **g44** | | 1R2B2 |  |  | 10 | 12 | B | R | **X** |  |  |
| **r52** | | 1R2B2 |  |  | 12 | 12 | R | R | **✓** |  |  |
| **g54** | | 2R2B2 |  |  | 3 | 3 | B | B | **✓** |  |  |
| **y20** | | 2R2B2 |  |  | 4 | 4 | R | B | **X** |  |  |
| **y55** | | 2R2B2 |  |  | 5 | 5 | B | B | **✓** |  |  |
| **w64** | | 2R2B2 |  |  | 11 | 12 | R | B | **X** |  |  |
|  | |  |  |  |  |  |  |  |  |  |  |

^1^Bees were recorded as having a preference for a box-opening behavioural variant if they performed this variant ≥60% of the time throughout the experiment. Bees that performed neither behavioural variant ≥60% of the time by the end of the experiment were classed as having no preference. ^2^Among bees with a preference for either behavioural variant, this preference was classed as ‘strong’ when the preferred variant was performed ≥80% of the time. It was classed as ‘weak’ when it was performed ≥60% but <80% of the time. As the sample sizes for non-proficient learners were small, no preferences or strength of preferences were assigned.
