# Supplementary material for: Bumblebees acquire alternative puzzle-box solutions via social learning
Source: PLoS Biol. 2023 Mar 7;21(3):e3002019. doi: 10.1371/journal.pbio.3002019 (PMC9990933; doi:10.1371/journal.pbio.3002019)
Supplement: S8 Table — (DOCX) [file pbio.3002019.s013.docx]

**Appendix Table 8. Results of linear mixed-effects model to assess the effect of time on learner preference**

| **A.** | | | | | | | |
| --- | --- | --- | --- | --- | --- | --- | --- |
| **Model** | **Random factor: Bee ID** | | | | | | |
| **Code** | responsevar ~ day.f + (1 \| beeID.f) | | | | | | |
|  | | **Sum Sq** | **Mean Sq** | **NumDF** | **DenDF** | **F value** | **Pr(>F)** |
| **Day.f** | | 0.06 | 0.06 | 1 | 11 | 0.5789 | 0.4627 |
| **AIC** | | 31.05 |  |  |  |  |  |
| **B.** | | | | | | | |
| **Model** | **Random factor: Population ID** | | | | | | |
| **Code** | responsevar~day.f +(1\|populationID.f) | | | | | | |
|  | | **Sum Sq** | **Mean Sq** | **NumDF** | **DenDF** | **F value** | **Pr(>F)** |
| **Day.f** | | 0.06 | 0.06 | 1 | 21 | 0.6260 | 0.4377 |
| **AIC** | | 26.17^#^ |  |  |  |  |  |
| **C.** | | | | | | | |
| **Model** | **Random factor: Bee ID nested within Population ID** | | | | | | |
| **Code** | responsevar~day.f +(1\|populationID.f/beeID.f) | | | | | | |
|  | | **Sum Sq** | **Mean Sq** | **NumDF** | **DenDF** | **F value** | **Pr(>F)** |
| **Day.f** | | 0.06 | 0.06 | 1 | 21 | 0.6260 | 0.4377 |
| **AIC** | | 28.17 |  |  |  |  |  |

^#^The selected model used for analysis, with the lowest AIC. There was one categorical fixed effect: one within-subjects factor “day” (day 1, day x; where day 1 was the day an individual met the learning criteria and day x was the last day they were recorded performing box-opening. Individuals only active on one day (n=1) were excluded, leaving n=12 (n=8 from population 1R2B2 and n=4 from 2R2B2). The response variable was the proportion of box-opening behaviour that was of the blue-pushing behavioural variant.
