# Supplementary material for: Bumblebees acquire alternative puzzle-box solutions via social learning
Source: PLoS Biol. 2023 Mar 7;21(3):e3002019. doi: 10.1371/journal.pbio.3002019 (PMC9990933; doi:10.1371/journal.pbio.3002019)
Supplement: Attachment — Submitted filename: 12012023 Bridges et al 2023 PLoS Biol Response to Reviewers.docx [file pbio.3002019.s014.docx]

**Response to Reviewers**

*Dear all,*

*Thank you so much to all reviewers for providing your thoughts on our manuscript, and for the chance to revise the article. Here, we address each of your points individually, with our responses highlighted in italics, and the line numbers provided correspond to those present in the “revised manuscript with changes highlighted” document. We believe that these changes have strengthened our manuscript, and hope that you will find the revised version suitable for publication.*

*Alice Bridges, on behalf of all authors*

***Academic editor comments***

A very interesting and timely study of cultural transmission in bumble bees. Thus far, small-scale experiments indicated social learning in insects, but this is the first experiment on social transmission in a more naturalistic setting with many participants and observers, i.e. the sort of context in which social learning will be adaptive. It will be good to mention that the two-action technique employed here derives from Andy Whiten’s primate studies with artificial fruits (ref. 25 Whiten Mesoudi 2008 rather than Aplin 2015, method also used in Whiten’s panpipe study in Nature of 2005 and the vervet monkey study by Erica vd Waal). Reviewer #1 urges to mention these studies, not just to explain where your methodology comes from but also to suggest a potential similarity in mechanisms between social learning in primates and insects.

*Thank you for these recommendations – we have now cited all these articles in the manuscript (Whiten Mesoudi 2008: Line 65, Whiten et al. 2005: Line 129, van de Waal et al. 2013/2017: Line 569)*

The whole idea of insect culture (wasps/bees) was already brought up by Kinji Imanishi in his 1952 essay, which forms the foundation of modern animal culture studies. The authors rightly mention Darwin’s thoughts in this regard, but should also read and mention Imanishi's classic.

*Thank you very much for bringing this to our attention: we have now added the reference to Imanishi’s work alongside relevant discussion (Lines 745-746).*

Both reviewers feel that the experiments conclusively demonstrate social learning. I commend the authors for their clear description of the methods and their convincing results. The fact that in the control settings some bees managed to learn how to access the sugar water is not a good argument against social transmission, which the experimental conditions clearly demonstrate, with opening following the model’s specific actions and not the alternative solution.

*Thank you for these encouraging comments: we agree, and hope that readers will come to a similar conclusion.*

The comparison with milk bottle opening by tits is not the best, as this habit may have spread without actual observational learning from others.

*We have removed this example entirely as part of our restructuring of our article’s focus away from culture and towards social learning.*

Apart from the many detailed suggestions and questions from both reviewers, which the authors should address in their reply and revision, there is the issue of how far to push the comparison with human culture (reviewer #2). It’s true that the term “culture” is now increasingly used in animal studies for any socially learned habit, skill, or behavioral tendency. In its more narrow sense, “culture” refers to systematic behavioral differences between groups or populations, i.e. behavioral differences produced by social learning, which set the behavior of group A apart from that of group B even if they live in the same environment. Social learning has the potential of producing intergroup variability, but this is not the focus of this study and not demonstrated here. In this sense, I’d be careful with the implication of culture.

*Thank you for your suggestion. We have extensively restructured our introduction and discussion sections to ensure that the paper focuses entirely on the social learning aspect, and only bring the idea of non-human culture in towards the end (Line 682 onwards)*

When reviewer #2 further suggests that cultures involve beliefs, we may be entering a discussion that we usually avoid in animal studies. My view is that the term “culture” can be used in the general sense (i.e. evidence of social learning), but that certain caveats need to be added to make clear that the more narrow sense of the term does not apply. Some contextualization of the term is needed and its presence in the title is perhaps also not warranted. I agree with reviewer #2 that to focus your writing on social learning and the spreading of habits, which is already spectacular enough, will be the best solution.

*As suggested, we have removed the word term “culture” from the title and rephrased it as “Bumblebee colonies (Bombus terrestris) acquire alternative puzzle-box solutions via social learning”.*

*Furthermore, where we introduce the concept of culture in the discussion, we have provided a full definition (Lines 742-745). We would like to thank everyone again for their thoughtful and critical comments on our manuscript, and hope that our revised manuscript will be acceptable for publication.*

***Reviewer#1***

This paper reports on an open-diffusion experiment of a novel artificial foraging technique. Its results are both convincing and newsworthy. Some 15 years ago, when these same patterns were revealed in chimpanzees, with brains thousands of times larger and far more sophisticated, many observers greeted them with disbelief. The great tits were already received more favorably. Now, the same pattern is found in humble bumblebees. This paper should reverberate among scholars of behavioral biology and human evolution, and provides much food for thought about the importance of sociality in local adaptation.

I have very few comments on the study itself. It seems to be perfect: it uses an established and tried design, and every step is clearly documented; the paper is also well-written and easy to follow. (I cannot judge whether the statistics are fully up to snuff, but did not see any obvious problems). 

Nonetheless, I propose to place these results a bit more into the broader historical and taxonomic context, which could lead to the recognition of broad principles of cultural transmission.

*Thank you so much for taking the time to review our manuscript so thoroughly, and many thanks again for your enthusiastic comments. We now discuss the chimpanzee results (Lines 126-129) and vervet monkey results (Lines 566-569) in greater detail and compare them explicitly with our results (Line 570 onwards). We have restructured the paper to focus more on social learning, and now primarily bring the culture discussion in towards the end, which we think keeps the relevant information closer together and provides a clearer flow of “thought” for the paper.*

L 49+           There is very little evidence for this effect on speciation, apart from the killer whale story, and I doubt anyone will seriously entertain the possibility that this is relevant to social insects. One way to avoid readers from overgeneralizing is to replace "also" with "even" in L 49. This is especially relevant because the next item (Baldwin effect) is returned to later and is far more central to the paper.

*Thank you: we have actually removed this sentence to avoid the potential for overgeneralizing.*

L 60+           This suggests we will see a lot more on this in the discussion, but there the development is a bit unfinished. I think it is worthwhile enhancing that discussion (see below).

*At this point, the Baldwin effect and the potential links between social learning and the behavioural repertoires of social insects remain very theoretical, and we only seek at this point to highlight this possibility and encourage future investigation, as it is indeed beyond the scope of this paper. We have reworded this to clarify our intent (L.N. 762 onwards).*

L 242+          Is there an explanation why the demonstrators had all stopped by day 6, whereas the learners continued? Had they aged or died? To prevent naïve questions, it may be useful to give some background information in the introduction about bumblebee forager ages, because I assume this is very different from honey bees.

*You are correct: the demonstrators had either ceased their foraging activity and taken up other “jobs” in the nest (their “career choices” are more flexible than honeybees and are not necessarily age-dependent – see Goulson 2003 Bumblebee behaviour and ecology, Oxford University Press) or they had died. Individual bumblebee workers can live for up to 2 months after emergence from the pupa, depending on the species (see Smeets & Duchateau 2003 Longevity of Bombus terrestris workers (Hymenoptera: Apidae) in relation to pollen availability, in the absence of foraging. Apidologie, , 34 (4), pp.333-337), but not all this time is spent foraging, and foraging does tend to occur later in life (Goulson 2003). When selecting demonstrators to train, we aimed to pick the “best”, most reliable foragers to speed up the process: it is likely that these had already been foraging for some time and may therefore have tended to be older. We have added clarification to L.N. 516-519, where this point is mentioned, and provided background information in the introduction (L.N. 55-58).*

L 257+          That in expt 3 individuals with very different preferences coexisted in the same group (linked to the presence of two types of demonstrators) suggests that there is no 'social pressure' to conform (hard to imagine anyway in bumblebees), and therefore that the tenacity of some preferences are based on individual tendencies (e.g. persistence of a learned preference due to habit formation: if it ain't broke don't fix it plus avoidance of unnecessary cognitive load). The difference with the presence of a social effect in various primate studies (after Whiten also work by Luncz and van de Waal) may be more apparent than real because the presence of different types of demonstrators may not trigger the social effect. Still, some discussion of this point may be worthwhile: how far does the simple explanation for persistence (habit formation) go in explaining all observed patterns (see also next comments)?

*Thank you for this suggestion: we have added some discussion of these points (habit formation, cost of switching to new behaviours) in the discussion (Lines 672-674). However, we think that further work would need to be done to say anything more concrete about how much either of these might explain the persistence of preferences (and resultant group level preferences).*

L 278+          A bit of history on the culture debate in biology may be useful here (cf. L81). This study's design was identical to that of Whiten et al. (2005, Nature), which got the ball rolling on the persistence and conformity features, with the latter perhaps better called within-group uniformity (see also Whiten at al. (2007, Current Biology) on the persistence). The great tit work then followed up with even more convincing results (larger samples, naturalistic conditions), as did van de Waal's work on vervet monkeys. Now we see these same features in social insects. I bring this up (I am not Andrew Whiten), because it may be worth stressing these strong similarities, because they point to shared (and thus potentially homologous) features of social learning with an exceptionally deep history.

*Thank you for this advice. We have now added references to these studies and relevant discussion as follows: Whiten et al. 2005: Line 129, van de Waal et al. 2013/2017: Line 569. We draw explicit comparisons in the discussion (Line 570 onwards).*

L 290+          This same result also held in the original Whiten et al. panpipes study, and more interestingly in the vervet study of van de Waal et al. (2017, Am J Primatol), which noted that females were shown to be quite persistent in their preferences, even when they, being subordinate, had only limited access to the group's preferred color, and therefore considerable experience with the other one (which was no longer distasteful), yet, after splitting from the main group and getting uncontested access, strongly preferred the original color. Again, this may suggests shared mechanisms, which are worth extracting (and then explaining).

*Thank you for this suggestion: discussion of this article and mention of these potential parallels has been added (Line 569 onwards).*

L 297+          This length of the discussion can perhaps be reduced by stressing more clearly in the introduction (L 72+) that the design of the study, which combines the two-action method  (made the gold standard by Jeff Galef), with training two different models along with a control group without any trained models, is exactly the kind that exclude non-social alternative explanations (massive parallel innovation), because of the clustering of the same action as that performed by the demonstrator, even if both actions are equally feasible.

*Thank you for your suggestion. We have added further emphasis on the study design in the introduction (Line 125 onwards) and have therefore been able to cut the length of the discussion.*

L 385+          This speculation may seem really plausible in light of the Robinson & Barron paper, but when cultural transmission fails to take a hold due to short lives and breaks in transmission due to seasonal or frequent colony die-offs, this needs to be developed far more specifically. Perhaps this is mainly a task for the future, but it may be worth mentioning that the R&B idea for the origin of 'instinct', if it relies on social learning rather than another mechanism, requires quite specific conditions. Related to this, the speculation on L 378+ on the kinds of species in which such a Baldwin process could have occurred leads to the phylogenetic prediction that the ancestors of species in which the conditions are currently not favorable for this kind of process had ancestors where these conditions held, so that the instincts could evolve.

*We accept (and explicitly highlight) that this part of the paper is mostly speculation, and our aim in including it was to highlight this possibility and encourage future investigation, as it is indeed beyond the scope of this paper. We have reworded this to clarify our intent (Lines 762 onwards).*

L 393+  I could not find any mention of colony sizes in the main text (it can perhaps be reconstructed by parsing the appendix tables -- which most readers won't do). Having that information, even if only as a ballpark figure, makes it easier to interpret the results (e.g. Figure 5 shows specific individuals, but were they the majority, or just a tiny proportion of the these groups?). This could be useful information.

*It is quite difficult to provide meaningful estimates of colony size for this experiment. There was continuous turnover, with new bees emerging and old bees dying. On any given day, only a small percentage of the total worker number of the nest would be actively foraging, and many bees would never emerge to forage at all (and so were essentially “superfluous” to the experiment because they were doing other jobs in the hive. Others would enter the flight arena but remain sitting in the corners off-camera, not interacting with the boxes at all. We thus focus our analysis on those individuals that did engage with the puzzle boxes.*

L 422           Figure 5 should be Figure 4. (Again L 429)

*Thank you for spotting this – we have now fixed these figure references to Fig. 4.*

***Reviewer#2***

This manuscript by Bridges and colleagues continues the very interesting work of Chittka's team on social learning in invertebrates. First of all I will first state that I absolutely love the work conducted in this lab, and I think it has clearly changed our way of thinking about invertebrates and their abilities, particularly with respect to social learning.

*Thank you very much for these encouraging words! And thank you so much for your thorough and extensive comments on our manuscript, which we believe will substantially improve it.*

This being said, I very much think that the current manuscript tries way too much to connect to culture, maybe as to connect to the 'sexy topic', and that by doing so, it weakens its own strength, which is social learning. Yes, the open diffusion and double seeding have been used by prominent authors as evidence of culture. Yet, it was in conjunction with other data, especially field data, showing behavioral diversity. On their own, these paradigms do not prove culture in any meaningful ways. In fact, one of the most influential study in the last decade using this paradigm (Aplin et al. 2015), is arguably in a
'non-cultural' species.

*Thank you for this advice – we agree that since we do not show such variation in the field, we should tone down the references to culture. We have reworded our title to remove reference to culture as well. Our study merely aims to investigate whether social learning in bees might take on forms that would, at least in theory, suggest that culture-like phenomena would be plausible. We do agree with you that it’s best not overreach in terms of the study’s aims, and have made this clear throughout.*

So, I have to say I'm very split about the way the manuscript is written, rather than about its results, which are very interesting, and very convincing. I would hope the authors reshape the manuscript towards social learning and showing the parallels with other famous studies, opening the discussion on whether this really shows that the seeds of culture are there or not, rather than upholding an elusive cultural argument, which at times (see below my comments) really feels forced (and easy to attack). I don't think this diminishes the value of the manuscript, which should eventually be published, and I also think these are very important data for the cultural debate (but not in the way the authors currently present it). My comments follow the manuscript.

*See previous comment (and also our reply to the editor) – we have taken this comment on board in full and present the discussion of culture largely as an afterthought late in the paper.*

L40-41: I'm not sure milk-bottle opening is really accepted as an example of culture in the sense that we sadly do not have evidence for social learning, which is princeps to culture. So it is instrumental to discussions of culture, clearly, but it is also a cautionary tale because we cannot show evidence of social learning (see for example the old chapters by Galef and collaborators). In that sense, the evidence for nectar robbing (which I did not know, so thank you for introducing me to it!) is stronger because there is evidence of social learning. 

In general, this point of details underlines quite the feeling I got from reading the intro: it is well written, and documented, but it tries too much to make the case for the 'culture' story, rather than remain focused on the strong empirical evidence for social learning, which, to me, have been notably brought convincingly shown by the present team. Yet, the framing of the introduction seems like "look, it's not another study of social learning done a different way, it's culture'. And I am not completely sure it is.

*Thank you for your comments. We have removed reference to milk-bottle opening throughout the article, as well as reframing the article to focus on social learning over culture. Mention of the possibility of culture is now brought in in the discussion section rather than being the overarching story of the paper from the beginning.*

L66-68: Yes, the open diffusion paradigm is clearly the focus of this paper. However, the fact that it is often being used to talk about culture, especially in cultural species such as chimpanzees does not make it the gold standard to 'show culture'. For example, often Lucy Aplin's fantastic work is cited in support of culture even though tits do not display any known cultural traits in the wild! I think it would be beneficial for the paper to clearly mark this difference. (and in a way, we come down to the same result at the end: the tits and the bees could have the potential for culture, in that they will converge on a certain solution, yet these findings do not appear to go beyond the lab/field work, and the survival of the trait is artificially maintained by the researchers).

*Thank you for these points – we believe we addressed most of these already in our responses thus far. An important point of our work is, as you state, B. terrestris is unlikely to show this type of culture in the wild. In fact, they have even less reason than tits to do so, yet they still appear capable of it if given opportunity.*

Results:
 
**Experiment 1**
Unless I'm mistaken, there is a crucial bit that appears in Table 2 and in the Appendix Table 1 but is not mentioned in the text: the fact that the models appear to account for a large amount of realized trials (around half or more). I think this would be a meaningful information to add because it also shows the 'observers' have many trials to learn from.

*Thank you for making this point: we have changed these tables to show the demonstrator behavioural data in place of the overall colony behavioural data for clarity.*

**Experiment 2**
L134-135: Echoing what I just wrote above, the bees that had demonstrators present also had way more trials to learn from. That does not render the results any less interesting, but I think a thorough social network/observation analysis should be presented, especially given the quality of the data presented in Appendix Table 1, which seem unexplored (unless the authors have another manuscript in preparation on this specific topic of spread).

*Thank you for your suggestion. We would have liked to perform network-based diffusion analysis, but this was not possible in our setup because any bee could potentially view any action performed by another bee from anywhere inside the arena.*

L150-51: Do we have evidence that the behavior remained 'fixed' in the individuals past the 12 days (I guess, for bees, it's possible to actually test the 'lifetime' claim?)

*Unfortunately, we did not test the bees beyond the 12 days maximum diffusion time. However, we did see several individuals maintaining their behaviour and their preferences for long spans during the experiment (b18 Fig. 5A, from day 2-12, g58 Fig. 5A, day 2-7, g56 Fig. 5B, day 2-10) and none seemed to change their preference. The lifetime claim in bees would, in theory, be possible to test due to their short lifespans, but bees spend only a short window of this lifespan (3-6 weeks) foraging, so these numbers are probably not far off.*
 
L160: Is it 'social learning' that is crucial, or 'social influence' under the form of practicing the behavior (I would here think of Claudio Tennie's misty framework of ZLS, with the behavior being somehow provoked by others' presence, but with little social learning).

*We have reworded this sentence slightly to allow for either (the presence of social information was crucial) – see Line 229.*

L162-179: To me, this is the real strength of the paper: replicating the findings in chimps, vervets & tits!

*Thank you for this encouragement – we agree. We hope that our modifications (Line 569 onwards) will help emphasize this finding over others.*

L211-214: I am not completely clear about this statement: in control group C4, it is actually the observers that performed most of the trials by the end of the experimental schedule. Granted it's one out of the 4 control groups, but this suggests that we cannot exclude that the behavior would be seeded. In contrast, in the experimental groups, it remains a 50/50 with the observers vs total trials. I am curious what would happen if the demonstrators would be removed. Was such experiment done?

*Unfortunately, we did not perform these experiments, and these questions would likely be best answered with a transmission chain paradigm (potentially combined with open diffusion, as in Danchin E, Nöbel S, Pocheville A, Dagaeff AC, Demay L, Alphand M, Ranty-Roby S, van Renssen L, Monier M, Gazagne E, Allain M, Isabel G. Cultural flies: Conformist social learning in fruitflies predicts long-lasting mate-choice traditions. Science. 2018  Nov 30;362(6418):1025-1030). As this paper is already lengthy, we believe this is best saved for follow-up studies, of which we hope to publish many to properly explore all elements of this topic. However, we do have unpublished data showing string-pulling behaviour being transmitted first throughout one colony from a trained demonstrator, and then from the learners of this colony to an entirely naïve colony. We hope to publish these data in future, but we felt that including data from a wholly different paradigm in this paper would confuse our narrative.*
 
Experiment 3
L242-246: "Strikingly, all demonstrators in this population had ceased their activity by day 6; all box-opening during the remaining six days was performed by observers alone (Appendix Table 5). Four new individuals reached the learning threshold after day six in this colony, suggesting that they may have learned from the previous generation of learners rather than the demonstrators (Table 3)."

 Do we have evidence that these 4 individuals did not watch the initial demonstrators? Or that there is a clear bias towards looking at the previous generation of learners? Once again, it seems the 'nice story' explanation is pushed. Sure, it makes a nice story and it would be really great if true, but I assume the authors have the data to actually explored that (e.g. carefully curated video analysis, or something like machine-learning based such as DLC could do)?

*We do not have clear evidence that the observers did not watch these demonstrators (and indeed the video data we generated would not have guaranteed this even if we had analysed every bee’s location on a frame-by-frame basis, as there were some areas that were off camera). We did qualify the highlighted statement with “suggested” and “may”, and are not attempting to make an assertion either way, but have reworded it to try and make this clearer (Lines 512-514). This would be something we would be really interested in looking at in the future, but would require the design of entirely new set-ups and/or the use of a transmission chain design, and is probably beyond the scope of this already lengthy paper.*
 
L271-274: I think this is a result that should be discussed in more details. Indeed, if the new learners form their own preference, then the 'tradition' side of the seeding is not really there, and hence the roots for cultural behavior to be maintained even less. 

*Thank you for raising this. We agree, and have discussed the possibility that local variation would be less likely to arise in the discussion (Lines 670-672) The bees did form their individual preferences in the multiple-demonstrator experiment, but in the single demonstrator experiment, they all copied the demonstrator’s choice. It may be that there is certain proportion of one variant being demonstrated over another, the observers acquire it over the other. Further work in the vein of Danchin et al’s fruit fly work (2018) could investigate this further.*

**Discussion**
 
L283: I think the above lines (271-274) directly contradict the 'persist' part of the sentence. I would remove it, or strongly dilute the sentence.

*Thank you for the suggestion: we have removed “persist” from this sentence (Line 559)*

L289-296: I think the authors should refer to the work of Erica van de Waal too. Especially considering some dominated individuals who reverted to the group alternative when they had the opportunity (ie, when there was no monopolization by the dominant individuals).

*Thank you for this suggestion. We agree, and have added reference to van de Waal’s work in vervet monkeys (Line 569).*

L297-310: I completely agree. But this is the usual straw man argument of 'pure social learning' theorists.

*Thank you – we have condensed this section as part of our reworked discussion, referring more strongly to our study design, which aims to exclude alternative non-social explanations*.

L317-318: Do we know that bees have 'attention'? I'm just asking because this is a very cognitively loaded word thrown into a largely cognition-free manuscript.

*Yes: please see the following references:*

*Spaethe, J., Tautz, J. & Chittka, L. (2006). Do honeybees detect colour targets using serial or parallel visual search? The Journal of Experimental Biology, 209: 987-993*

*Nityananda V, Chittka L. 2015 Modality-specific attention in foraging. bumblebees. R. Soc. open sci. 2:150324*

*Morawetz L., Spaethe J.; Visual attention in a complex search task differs between honeybees and bumblebees. J Exp Biol 15 July 2012; 215 (14): 2515–2523*

*However, we have removed the word attention for ease (L.N. 633).*

L334-347: Again, I think that shows the strength of the data, and to me, what should be the focus of the paper!

*Thank you: as discussed above, we have made efforts to re-frame the paper away from culture and towards social learning, focusing on what our core data shows.*

L352-358: Once again, it feels the authors try to tie the results t

o culture in humans, with stochasticity leading to shifts or change of culture. I think that is completely unnecessary, especially because we are here talking about beliefs etc. Unless the authors want to engage in a debate on whether bees have views on the world, which is beyond the scope of this paper, I would suggest refraining from this easy -to-attack angles.

*We agree that this sentence may have distracted from the main point of the paper, thus have removed it to avoid confusion.*

L365: "at least in theory". Once again, a lot of speculation to make a point. The question is rather: why not test it?

*Thank you for raising this: we have removed this sentence as we can see how it might distract from the main points of the paper.*

L367 onward: after having pushed the idea of culture all the way through the manuscript, the authors then acknowledge that it is difficult to see it as a real possibility because of the socio-ecology of the bee species they study. I think that acknowledgement itself is more than welcome. In fact I think it would be more interesting to be up front about it in the introduction, and say that while the authors are looking for necessary components of culture, it may not really exist, because of the lifespan/socio-ecology. That would actually call for a good discussion on this very topic afterwards.

*Thank you for this suggestion. We believe that this concern should be addressed by our re-framing of our experiment throughout the manuscript, as discussed thus far.*
