# Supplementary material for: Bumblebees acquire alternative puzzle-box solutions via social learning
Source: PLoS Biol. 2023 Mar 7;21(3):e3002019. doi: 10.1371/journal.pbio.3002019 (PMC9990933; doi:10.1371/journal.pbio.3002019)
Supplement: Peer review history

# Editor report: Decision Letter 0

**DOI:** 10.1371/journal.pbio.3002019.r001

**Authors:** Roland G Roberts

17 Oct 2022

Dear Dr Bridges, 

Thank you for submitting your manuscript entitled "The establishment and transmission of novel foraging techniques indicates a capacity for culture in bumblebees (Bombus terrestris)" for consideration as a Research Article by PLOS Biology.

Your manuscript has now been evaluated by the PLOS Biology editorial staff, as well as by an academic editor with relevant expertise, and I'm writing to let you know that we would like to send your submission out for external peer review.

However, before we can send your manuscript to reviewers, we need you to complete your submission by providing the metadata that is required for full assessment. To this end, please login to Editorial Manager where you will find the paper in the 'Submissions Needing Revisions' folder on your homepage. Please click 'Revise Submission' from the Action Links and complete all additional questions in the submission questionnaire.

Once your full submission is complete, your paper will undergo a series of checks in preparation for peer review. After your manuscript has passed the checks it will be sent out for review. To provide the metadata for your submission, please Login to Editorial Manager (https://www.editorialmanager.com/pbiology) within two working days, i.e. by Oct 19 2022 11:59PM.

If your manuscript has been previously peer-reviewed at another journal, PLOS Biology is willing to work with those reviews in order to avoid re-starting the process. Submission of the previous reviews is entirely optional and our ability to use them effectively will depend on the willingness of the previous journal to confirm the content of the reports and share the reviewer identities. Please note that we reserve the right to invite additional reviewers if we consider that additional/independent reviewers are needed, although we aim to avoid this as far as possible. In our experience, working with previous reviews does save time. 

If you would like us to consider previous reviewer reports, please edit your cover letter to let us know and include the name of the journal where the work was previously considered and the manuscript ID it was given. In addition, please upload a response to the reviews as a 'Prior Peer Review' file type, which should include the reports in full and a point-by-point reply detailing how you have or plan to address the reviewers' concerns. 

During the process of completing your manuscript submission, you will be invited to opt-in to posting your pre-review manuscript as a bioRxiv preprint. Visit http://journals.plos.org/plosbiology/s/preprints for full details. If you consent to posting your current manuscript as a preprint, please upload a single Preprint PDF. 

Feel free to email us at plosbiology@plos.org if you have any queries relating to your submission.

Kind regards,

Roli Roberts

Roland Roberts, PhD

Senior Editor

PLOS Biology

rroberts@plos.org

# Peer review: Decision Letter 1

**DOI:** 10.1371/journal.pbio.3002019.r002

**Authors:** Roland G Roberts

13 Dec 2022

Dear Dr Bridges,

Thank you for your patience while your manuscript "The establishment and transmission of novel foraging techniques indicates a capacity for culture in bumblebees (Bombus terrestris)" went through peer-review at PLOS Biology. Your manuscript has now been evaluated by the PLOS Biology editors, an Academic Editor with relevant expertise, and by two independent reviewers. We had recruited a third reviewer, but they were not able to deliver their comments in a timely fashion.

In light of the reviews, which you will find at the end of this email, we are pleased to offer you the opportunity to address the comments from the reviewers in a revision that we anticipate should not take you very long. IMPORTANT: Please note that the Academic Editor has kindly also supplied some additional comments (see the foot of this email) which you may find useful. We will then assess your revised manuscript and your response to the reviewers' comments with our Academic Editor aiming to avoid further rounds of peer-review, although might need to consult with the reviewers, depending on the nature of the revisions.

We expect to receive your revised manuscript within 1 month. Please email us (plosbiology@plos.org) if you have any questions or concerns, or would like to request an extension. 

At this stage, your manuscript remains formally under active consideration at our journal; please notify us by email if you do not intend to submit a revision so that we withdraw the manuscript.

**IMPORTANT - SUBMITTING YOUR REVISION**

Your revisions should address the specific points made by each reviewer. Please submit the following files along with your revised manuscript:

1. A 'Response to Reviewers' file - this should detail your responses to the editorial requests, present a point-by-point response to all of the reviewers' comments, and indicate the changes made to the manuscript. 

*NOTE: In your point-by-point response to the reviewers, please provide the full context of each review. Do not selectively quote paragraphs or sentences to reply to. The entire set of reviewer comments should be present in full and each specific point should be responded to individually.

You should also cite any additional relevant literature that has been published since the original submission and mention any additional citations in your response. 

2. In addition to a clean copy of the manuscript, please also upload a 'track-changes' version of your manuscript that specifies the edits made. This should be uploaded as a "Revised Article with Changes Highlighted " file type. 

*Resubmission Checklist*

When you are ready to resubmit your revised manuscript, please refer to this resubmission checklist: https://plos.io/Biology_Checklist

To submit a revised version of your manuscript, please go to https://www.editorialmanager.com/pbiology/ and log in as an Author. Click the link labelled 'Submissions Needing Revision' where you will find your submission record. 

Please make sure to read the following important policies and guidelines while preparing your revision:

*Published Peer Review*

Please note while forming your response, if your article is accepted, you may have the opportunity to make the peer review history publicly available. The record will include editor decision letters (with reviews) and your responses to reviewer comments. If eligible, we will contact you to opt in or out. Please see here for more details:

https://blogs.plos.org/plos/2019/05/plos-journals-now-open-for-published-peer-review/

*PLOS Data Policy*

Please note that as a condition of publication PLOS' data policy (http://journals.plos.org/plosbiology/s/data-availability) requires that you make available all data used to draw the conclusions arrived at in your manuscript. If you have not already done so, you must include any data used in your manuscript either in appropriate repositories, within the body of the manuscript, or as supporting information (N.B. this includes any numerical values that were used to generate graphs, histograms etc.). For an example see here: http://www.plosbiology.org/article/info%3Adoi%2F10.1371%2Fjournal.pbio.1001908#s5

*Blot and Gel Data Policy*

We require the original, uncropped and minimally adjusted images supporting all blot and gel results reported in an article's figures or Supporting Information files. We will require these files before a manuscript can be accepted so please prepare them now, if you have not already uploaded them. Please carefully read our guidelines for how to prepare and upload this data: https://journals.plos.org/plosbiology/s/figures#loc-blot-and-gel-reporting-requirements

*Protocols deposition*

To enhance the reproducibility of your results, we recommend that if applicable you deposit your laboratory protocols in protocols.io, where a protocol can be assigned its own identifier (DOI) such that it can be cited independently in the future. Additionally, PLOS ONE offers an option for publishing peer-reviewed Lab Protocol articles, which describe protocols hosted on protocols.io. Read more information on sharing protocols at https://plos.org/protocols?utm_medium=editorial-email&utm_source=authorletters&utm_campaign=protocols

Thank you again for your submission to our journal. We hope that our editorial process has been constructive thus far, and we welcome your feedback at any time. Please don't hesitate to contact us if you have any questions or comments.

Sincerely,

Roli Roberts

Roland Roberts, PhD

Senior Editor

PLOS Biology

rroberts@plos.org

----------------------------------------------------------------

REVIEWERS' COMMENTS:

Reviewer #1:

This paper reports on an open-diffusion experiment of a novel artificial foraging technique. Its results are both convincing and newsworthy. Some 15 years ago, when these same patterns were revealed in chimpanzees, with brains thousands of times larger and far more sophisticated, many observers greeted them with disbelief. The great tits were already received more favorably. Now, the same pattern is found in humble bumblebees. This paper should reverberate among scholars of behavioral biology and human evolution, and provides much food for thought about the importance of sociality in local adaptation.

I have very few comments on the study itself. It seems to be perfect: it uses an established and tried design, and every step is clearly documented; the paper is also well-written and easy to follow. (I cannot judge whether the statistics are fully up to snuff, but did not see any obvious problems). 

Nonetheless, I propose to place these results a bit more into the broader historical and taxonomic context, which could lead to the recognition of broad principles of cultural transmission.

L 49+ There is very little evidence for this effect on speciation, apart from the killer whale story, and I doubt anyone will seriously entertain the possibility that this is relevant to social insects. One way to avoid readers from overgeneralizing is to replace "also" with "even" in L 49. This is especially relevant because the next item (Baldwin effect) is returned to later and is far more central to the paper.

L 60+ This suggests we will see a lot more on this in the discussion, but there the development is a bit unfinished. I think it is worthwhile enhancing hat discussion (see below).

L 242+ Is there an explanation why the demonstrators had all stopped by day 6, whereas the learners continued? Had they aged or died? To prevent naïve questions, it may be useful to give some background information in the introduction about bumblebee forager ages, because I assume this is very different from honey bees. 

L 257+ That in expt 3 individuals with very different preferences coexisted in the same group (linked to the presence of two types of demonstrators) suggests that there is no 'social pressure' to conform (hard to imagine anyway in bumblebees), and therefore that the tenacity of some preferences are based on individual tendencies (e.g. persistence of a learned preference due to habit formation: if it ain't broke don't fix it plus avoidance of unnecessary cognitive load). The difference with the presence of a social effect in various primate studies (after Whiten also work by Luncz and van de Waal) may be more apparent than real because the presence of different types of demonstrators may not trigger the social effect. Still, some discussion of this point may be worthwhile: how far does the simple explanation for persistence (habit formation) go in explaining all observed patterns (see also next comments)?

L 278+ A bit of history on the culture debate in biology may be useful here (cf. L81). This study's design was identical to that of Whiten et al. (2005, Nature), which got the ball rolling on the persistence and conformity features, with the latter perhaps better called within-group uniformity (see also Whiten at al. (2007, Current Biology) on the persistence). The great tit work then followed up with even more convincing results (larger samples, naturalistic conditions), as did van de Waal's work on vervet monkeys. Now we see these same features in social insects. I bring this up (I am not Andrew Whiten), because it may be worth stressing these strong similarities, because they point to shared (and thus potentially homologous) features of social learning with an exceptionally deep history.

L 290+ This same result also held in the original Whiten et al. panpipes study, and more interestingly in the vervet study of van de Waal et al. (2017, Am J Primatol), which noted that females were shown to be quite persistent in their preferences, even when they, being subordinate, had only limited access to the group's preferred color, and therefore considerable experience with the other one (which was no longer distasteful), yet, after splitting from the main group and getting uncontested access, strongly preferred the original color. Again, this may suggests shared mechanisms, which are worth extracting (and then explaining).

L 297+ This length of the discussion can perhaps be reduced by stressing more clearly in the introduction (L 72+) that the design of the study, which combines the two-action method (made the gold standard by Jeff Galef), with training two different models along with a control group without any trained models, is exactly the kind that exclude non-social alternative explanations (massive parallel innovation), because of the clustering of the same action as that performed by the demonstrator, even if both actions are equally feasible.

L 385+ This speculation may seem really plausible in light of the Robinson & Barron paper, but when cultural transmission fails to take a hold due to short lives and breaks in transmission due to seasonal or frequent colony die-offs, this needs to be developed far more specifically. Perhaps this is mainly a task for the future, but it may be worth mentioning that the R&B idea for the origin of 'instinct', if it relies on social learning rather than another mechanism, requires quite specific conditions. Related to this, the speculation on L 378+ on the kinds of species in which such a Baldwin process could have occurred leads to the phylogenetic prediction that the ancestors of species in which the conditions are currently not favorable for this kind of process had ancestors where these conditions held, so that the instincts could evolve.

L 393+ I could not find any mention of colony sizes in the main text (it can perhaps be reconstructed by parsing the appendix tables -- which most readers won't do). Having that information, even if only as a ballpark figure, makes it easier to interpret the results (e.g. Figure 5 shows specific individuals, but were they the majority, or just a tiny proportion of the these groups?). This could be useful information.

L 422 Figure 5 should be Figure 4. (Again L 429)

Reviewer #2:

This manuscript by Bridges and colleagues continues the very interesting work of Chittka's team on social learning in invertebrates. First of all I will first state that I absolutely love the work conducted in this lab, and I think it has clearly changed our way of thinking about invertebrates and their abilities, particularly with respect to social learning. This being said, I very much think that the current manuscript tries way too much to connect to culture, maybe as to connect to the 'sexy topic', and that by doing so, it weakens its own strength, which is social learning. Yes, the open diffusion and double seeding have been used by prominent authors as evidence of culture. Yet, it was in conjunction with other data, especially field data, showing behavioral diversity. On their own, these paradigms do not prove culture in any meaningful ways. In fact, one of the most influential study in the last decade using this paradigm (Aplin et al. 2015), is arguably in a 'non-cultural' species. So, I have to say I'm very split about the way the manuscript is written, rather than about its results, which are very interesting, and very convincing. I would hope the authors reshape the manuscript towards social learning and showing the parallels with other famous studies, opening the discussion on whether this really shows that the seeds of culture are there or not, rather than upholding an elusive cultural argument, which at times (see below my comments) really feels forced (and easy to attack). I don't think this diminishes the value of the manuscript, which should eventually be published, and I also think these are very important data for the cultural debate (but not in the way the authors currently present it). My comments follow the manuscript. 

L40-41: I'm not sure milk-bottle opening is really accepted as an example of culture in the sense that we sadly do not have evidence for social learning, which is princeps to culture. So it is instrumental to discussions of culture, clearly, but it is also a cautionary tale because we cannot show evidence of social learning (see for example the old chapters by Galef and collaborators). In that sense, the evidence for nectar robbing (which I did not know, so thank you for introducing me to it!) is stronger because there is evidence of social learning. 

In general, this point of details underlines quite the feeling I got from reading the intro: it is well written, and documented, but it tries too much to make the case for the 'culture' story, rather than remain focused on the strong empirical evidence for social learning, which, to me, have been notably brought convincingly shown by the present team. Yet, the framing of the introduction seems like "look, it's not another study of social learning done a different way, it's culture'. And I am not completely sure it is.

L66-68: Yes, the open diffusion paradigm is clearly the focus of this paper. However, the fact that it is often being used to talk about culture, especially in cultural species such as chimpanzees does not make it the gold standard to 'show culture'. For example, often Lucy Aplin's fantastic work is cited in support of culture even though tits do not display any known cultural traits in the wild! I think it would be beneficial for the paper to clearly mark this difference. (and in a way, we come down to the same result at the end: the tits and the bees could have the potential for culture, in that they will converge on a certain solution, yet these findings do not appear to go beyond the lab/field work, and the survival of the trait is artificially maintained by the researchers).

Results:

Experiment 1

Unless I'm mistaken, there is a crucial bit that appears in Table 2 and in the Appendix Table 1 but is not mentioned in the text: the fact that the models appear to account for a large amount of realized trials (around half or more). I think this would be a meaningful information to add because it also shows the 'observers' have many trials to learn from.

Experiment 2

L134-135: Echoing what I just wrote above, the bees that had demonstrators present also had way more trials to learn from. That does not render the results any less interesting, but I think a thorough social network/observation analysis should be presented, especially given the quality of the data presented in Appendix Table 1, which seem unexplored (unless the authors have another manuscript in preparation on this specific topic of spread).

L150-51: Do we have evidence that the behavior remained 'fixed' in the individuals past the 12 days (I guess, for bees, it's possible to actually test the 'lifetime' claim?)

L160: Is it 'social learning' that is crucial, or 'social influence' under the form of practicing the behavior (I would here think of Claudio Tennie's misty framework of ZLS, with the behavior being somehow provoked by others' presence, but with little social learning).

L162-179: To me, this is the real strength of the paper: replicating the findings in chimps, vervets & tits! 

L211-214: I am not completely clear about this statement: in control group C4, it is actually the observers that performed most of the trials by the end of the experimental schedule. Granted it's one out of the 4 control groups, but this suggests that we cannot exclude that the behavior would be seeded. In contrast, in the experimental groups, it remains a 50/50 with the observers vs total trials. I am curious what would happen if the demonstrators would be removed. Was such experiment done?

Experiment 3

L242-246: "Strikingly, all demonstrators in this population had ceased their activity by day 6; all box-opening during the remaining six days was performed by observers alone (Appendix Table 5). Four new individuals reached the learning threshold after day six in this colony, suggesting that they may have learned from the previous generation of learners rather than the demonstrators (Table 3)."

Do we have evidence that these 4 individuals did not watch the initial demonstrators? Or that there is a clear bias towards looking at the previous generation of learners? Once again, it seems the 'nice story' explanation is pushed. Sure, it makes a nice story and it would be really great if true, but I assume the authors have the data to actually explored that (e.g. carefully curated video analysis, or something like machine-learning based such as DLC could do)?

L271-274: I think this is a result that should be discussed in more details. Indeed, if the new learners form their own preference, then the 'tradition' side of the seeding is not really there, and hence the roots for cultural behavior to be maintained even less. 

Discussion

L283: I think the above lines (271-274) directly contradict the 'persist' part of the sentence. I would remove it, or strongly dilute the sentence. 

L289-296: I think the authors should refer to the work of Erica van de Waal too. Especially considering some dominated individuals who reverted to the group alternative when they had the opportunity (ie, when there was no monopolization by the dominant individuals).

L297-310: I completely agree. But this is the usual straw man argument of 'pure social learning' theorists. 

L317-318: Do we know that bees have 'attention'? I'm just asking because this is a very cognitively loaded word thrown into a largely cognition-free manuscript.

L334-347: Again, I think that shows the strength of the data, and to me, what should be the focus of the paper!

L352-358: Once again, it feels the authors try to tie the results to culture in humans, with stochasticity leading to shifts or change of culture. I think that is completely unnecessary, especially because we are here talking about beliefs etc. Unless the authors want to engage in a debate on whether bees have views on the world, which is beyond the scope of this paper, I would suggest refraining from this easy -to-attack angles.

L365: "at least in theory". Once again, a lot of speculation to make a point. The question is rather: why not test it?

L367 onward: after having pushed the idea of culture all the way through the manuscript, the authors then acknowledge that it is difficult to see it as a real possibility because of the socio-ecology of the bee species they study. I think that acknowledgement itself is more than welcome. In fact I think it would be more interesting to be up front about it in the introduction, and say that while the authors are looking for necessary components of culture, it may not really exist, because of the lifespan/socio-ecology. That would actually call for a good discussion on this very topic afterwards.

COMMENTS FROM THE ACADEMIC EDITOR:

A very interesting and timely study of cultural transmission in bumble bees. Thus far, small-scale experiments indicated social learning in insects, but this is the first experiment on social transmission in a more naturalistic setting with many participants and observers, i.e. the sort of context in which social learning will be adaptive. It will be good to mention that the two-action technique employed here derives from Andy Whiten’s primate studies with artificial fruits (ref. 25 Whiten Mesoudi 2008 rather than Aplin 2015, method also used in Whiten’s panpipe study in Nature of 2005 and the vervet monkey study by Erica vd Waal). Reviewer #1 urges to mention these studies, not just to explain where your methodology comes from but also to suggest a potential similarity in mechanisms between social learning in primates and insects.

The whole idea of insect culture (wasps/bees) was already brought up by Kinji Imanishi in his 1952 essay, which forms the foundation of modern animal culture studies. The authors rightly mention Darwin’s thoughts in this regard, but should also read and mention Imanishi's classic.

Both reviewers feel that the experiments conclusively demonstrate social learning. I commend the authors for their clear description of the methods and their convincing results. The fact that in the control settings some bees managed to learn how to access the sugar water is not a good argument against social transmission, which the experimental conditions clearly demonstrate, with opening following the model’s specific actions and not the alternative solution. The comparison with milk bottle opening by tits is not the best, as this habit may have spread without actual observational learning from others.

Apart from the many detailed suggestions and questions from both reviewers, which the authors should address in their reply and revision, there is the issue of how far to push the comparison with human culture (reviewer #2). It’s true that the term “culture” is now increasingly used in animal studies for any socially learned habit, skill, or behavioral tendency. In its more narrow sense, “culture” refers to systematic behavioral differences between groups or populations, i.e. behavioral differences produced by social learning, which set the behavior of group A apart from that of group B even if they live in the same environment.

Social learning has the potential of producing intergroup variability, but this is not the focus of this study and not demonstrated here. In this sense, I’d be careful with the implication of culture. When reviewer #2 further suggests that cultures involve beliefs, we may be entering a discussion that we usually avoid in animal studies. My view is that the term “culture” can be used in the general sense (i.e. evidence of social learning), but that certain caveats need to be added to make clear that the more narrow sense of the term does not apply. Some contextualization of the term is needed and its presence in the title is perhaps also not warranted. I agree with reviewer #2 that to focus your writing on social learning and the spreading of habits, which is already spectacular enough, will be the best solution.

# Author comment: Author response to Decision Letter 1

**DOI:** 10.1371/journal.pbio.3002019.r003

16 Jan 2023

Attachment Submitted filename: 12012023 Bridges et al 2023 PLoS Biol Response to Reviewers.docx Click here for additional data file.

# Editor report: Decision Letter 2

**DOI:** 10.1371/journal.pbio.3002019.r004

**Authors:** Roland G Roberts

25 Jan 2023

Dear Alice,

Thank you for your patience while we considered your revised manuscript "Bumblebees (Bombus terrestris) acquire alternative puzzle-box solutions via social learning" for publication as a Research Article at PLOS Biology. This revised version of your manuscript has been evaluated by the PLOS Biology editors and the Academic Editor.

Based on our Academic Editor's assessment of your revision, we are likely to accept this manuscript for publication, provided you satisfactorily address the following data and other policy-related requests:

a) Please change your title to "Bumblebees acquire alternative puzzle-box solutions via social learning" (we think that as the Linnaean name appears prominently in the Abstract, it's superfluous in the Title)

b) Please address my Data Policy requests below; specifically, we need you to supply the numerical values underlying Figs 1CD, 2AB, 3AB, 4BCD, 5AB, S1AB, S2AB, S3ABC, either as a supplementary data file or as a permanent DOI’d deposition (e.g. as part of your Figshare deposition, which I understand currently only contains “raw” data, rather than the values that appear in the Figs).

c) Please cite the location of the data clearly in all relevant main and supplementary Figure legends, e.g. “The data underlying this Figure can be found in S1 Data” or “The data underlying this Figure can be found in https://doi.org/XXXX”

As you address these items, please take this last chance to review your reference list to ensure that it is complete and correct. If you have cited papers that have been retracted, please include the rationale for doing so in the manuscript text, or remove these references and replace them with relevant current references. Any changes to the reference list should be mentioned in the cover letter that accompanies your revised manuscript.

We expect to receive your revised manuscript within two weeks. 

To submit your revision, please go to https://www.editorialmanager.com/pbiology/ and log in as an Author. Click the link labelled 'Submissions Needing Revision' to find your submission record. Your revised submission must include the following:

- a cover letter that should detail your responses to any editorial requests, if applicable, and whether changes have been made to the reference list

- a Response to Reviewers file that provides a detailed response to the reviewers' comments (if applicable)

- a track-changes file indicating any changes that you have made to the manuscript. 

NOTE: If Supporting Information files are included with your article, note that these are not copyedited and will be published as they are submitted. Please ensure that these files are legible and of high quality (at least 300 dpi) in an easily accessible file format. For this reason, please be aware that any references listed in an SI file will not be indexed. For more information, see our Supporting Information guidelines:

https://journals.plos.org/plosbiology/s/supporting-information

*Published Peer Review History*

Please note that you may have the opportunity to make the peer review history publicly available. The record will include editor decision letters (with reviews) and your responses to reviewer comments. If eligible, we will contact you to opt in or out. Please see here for more details:

https://blogs.plos.org/plos/2019/05/plos-journals-now-open-for-published-peer-review/

*Press*

Should you, your institution's press office or the journal office choose to press release your paper, please ensure you have opted out of Early Article Posting on the submission form. We ask that you notify us as soon as possible if you or your institution is planning to press release the article.

*Protocols deposition*

To enhance the reproducibility of your results, we recommend that if applicable you deposit your laboratory protocols in protocols.io, where a protocol can be assigned its own identifier (DOI) such that it can be cited independently in the future. Additionally, PLOS ONE offers an option for publishing peer-reviewed Lab Protocol articles, which describe protocols hosted on protocols.io. Read more information on sharing protocols at https://plos.org/protocols?utm_medium=editorial-email&utm_source=authorletters&utm_campaign=protocols

Please do not hesitate to contact me should you have any questions.

Sincerely,

Roli

Roland Roberts, PhD

Senior Editor,

rroberts@plos.org,

PLOS Biology

------------------------------------------------------------------------

DATA POLICY:

You may be aware of the PLOS Data Policy, which requires that all data be made available without restriction: http://journals.plos.org/plosbiology/s/data-availability. For more information, please also see this editorial: http://dx.doi.org/10.1371/journal.pbio.1001797

Note that we do not require all raw data. Rather, we ask that all individual quantitative observations that underlie the data summarized in the figures and results of your paper be made available in one of the following forms:

1) Supplementary files (e.g., excel). Please ensure that all data files are uploaded as 'Supporting Information' and are invariably referred to (in the manuscript, figure legends, and the Description field when uploading your files) using the following format verbatim: S1 Data, S2 Data, etc. Multiple panels of a single or even several figures can be included as multiple sheets in one excel file that is saved using exactly the following convention: S1_Data.xlsx (using an underscore).

2) Deposition in a publicly available repository. Please also provide the accession code or a reviewer link so that we may view your data before publication. 

Regardless of the method selected, please ensure that you provide the individual numerical values that underlie the summary data displayed in the following figure panels as they are essential for readers to assess your analysis and to reproduce it: Figs 1CD, 2AB, 3AB, 4BCD, 5AB, S1AB, S2AB, S3ABC. NOTE: the numerical data provided should include all replicates AND the way in which the plotted mean and errors were derived (it should not present only the mean/average values).

IMPORTANT: Please also ensure that figure legends in your manuscript include information on where the underlying data can be found, and ensure your supplemental data file/s has a legend.

Please ensure that your Data Statement in the submission system accurately describes where your data can be found.

------------------------------------------------------------------------

DATA NOT SHOWN?

- Please note that per journal policy, we do not allow the mention of "data not shown", "personal communication", "manuscript in preparation" or other references to data that is not publicly available or contained within this manuscript. Please either remove mention of these data or provide figures presenting the results and the data underlying the figure(s).

------------------------------------------------------------------------

# Author comment: Author response to Decision Letter 2

**DOI:** 10.1371/journal.pbio.3002019.r005

1 Feb 2023

Attachment Submitted filename: 12012023 Bridges et al 2023 PLoS Biol Response to Reviewers.docx Click here for additional data file.

# Editor report: Decision Letter 3

**DOI:** 10.1371/journal.pbio.3002019.r006

**Authors:** Roland G Roberts

2 Feb 2023

Dear Alice,

Thank you for the submission of your revised Research Article "Bumblebees acquire alternative puzzle-box solutions via social learning" for publication in PLOS Biology. On behalf of my colleagues and the Academic Editor, Frans de Waal, I'm pleased to say that we can in principle accept your manuscript for publication, provided you address any remaining formatting and reporting issues. These will be detailed in an email you should receive within 2-3 business days from our colleagues in the journal operations team; no action is required from you until then. Please note that we will not be able to formally accept your manuscript and schedule it for publication until you have completed any requested changes.

Please take a minute to log into Editorial Manager at http://www.editorialmanager.com/pbiology/, click the "Update My Information" link at the top of the page, and update your user information to ensure an efficient production process.

PRESS: We frequently collaborate with press offices. If your institution or institutions have a press office, please notify them about your upcoming paper at this point, to enable them to help maximise its impact. If the press office is planning to promote your findings, we would be grateful if they could coordinate with biologypress@plos.org. If you have previously opted in to the early version process, we ask that you notify us immediately of any press plans so that we may opt out on your behalf.

We also ask that you take this opportunity to read our Embargo Policy regarding the discussion, promotion and media coverage of work that is yet to be published by PLOS. As your manuscript is not yet published, it is bound by the conditions of our Embargo Policy. Please be aware that this policy is in place both to ensure that any press coverage of your article is fully substantiated and to provide a direct link between such coverage and the published work. For full details of our Embargo Policy, please visit http://www.plos.org/about/media-inquiries/embargo-policy/.

Thank you again for choosing PLOS Biology for publication and supporting Open Access publishing. We look forward to publishing your study. 

Sincerely,

Roli

Roland G Roberts, PhD, PhD

Senior Editor

PLOS Biology

rroberts@plos.org
